# Use of CR-39 Dosimeters for the Imaging of Neutron Beam Profiles in the 100 keV–10 MeV Energy Range

**DOI:** 10.3390/s25185865

**Published:** 2025-09-19

**Authors:** Margherita Simoni, Leonardo Baldassarre, Carlo Cazzaniga, Laura Fazi, Mattia Gaboardi, Leandro Gemmiti, Maria Kastriotou, Matthew Krzystyniak, Anna Marsicano, Marco Martellucci, Triestino Minniti, Anna Prioriello, Roberto Senesi, Valentin Suteica, Giovanni Romanelli

**Affiliations:** 1 Dipartimento di Fisica and NAST Centre, Università degli Studi di Roma Tor Vergata, Via della Ricerca Scientifica 1, 00133 Roma, Italy; margherita.simoni@uniroma2.it (M.S.); laura.fazi@uniroma2.it (L.F.); triestino.minniti@uniroma2.it (T.M.); anna.prioriello@uniroma2.it (A.P.); roberto.senesi@uniroma2.it (R.S.); valentin.suteica@students.uniroma2.eu (V.S.); giovanni.romanelli@uniroma2.it (G.R.); 2 L.B. Servizi per le Aziende srl, Via Gabriele Paleotti 43, 00168 Roma, Italy; l.baldassarre@lbservizi.it (L.B.); l.gemmiti@lbservizi.it (L.G.); 3 ISIS Neutron and Muon Source, UKRI-STFC, Rutherford Appleton Laboratory, Harwell Campus, Didcot, Oxfordshire OX11 0QX, UK; carlo.cazzaniga@stfc.ac.uk (C.C.); maria.kastriotou@stfc.ac.uk (M.K.); matthew.krzystyniak@stfc.ac.uk (M.K.); anna.marsicano@stfc.ac.uk (A.M.); 4 Mardel Srl, Via Topino 35, 00199 Roma, Italy; m.martellucci@mardel.it; 5 Istituto di Struttura della Materia (ISM), Consiglio Nazionale delle Ricerche, Via del Fosso del Cavaliere 100, 00133 Rome, Italy

**Keywords:** CR-39, solid-state nuclear track detectors, neutron beam profile

## Abstract

We provide a beam shape characterization of the VESUVIO spectrometer, at the ISIS Neutron and Muon Source, employing CR-39 solid-state nuclear track detectors and combining techniques including optical and electron microscopy, as well as Monte Carlo transport simulations. In particular, we show, through comparison with irradiation with 14 MeV neutrons at the NILE Facility at ISIS, that the majority of defects on the etched surface of the dosimeters irradiated on VESUVIO were induced by neutrons with energies between 100 keV and 10 MeV. Our results were compared to previous characterizations of the VESUVIO beam shape performed in either the thermal or fast energy ranges, and we conclude that the VESUVIO beam has a constant shape from thermal-neutron energies up to 10 MeV, composed of an umbra (intensity above 90% of the maximum) with radius 1.1 cm, and surrounded by a penumbra (intensity above 1% of the maximum) that extends up to 2.5 cm.

## 1. Introduction

**Figure 1:**
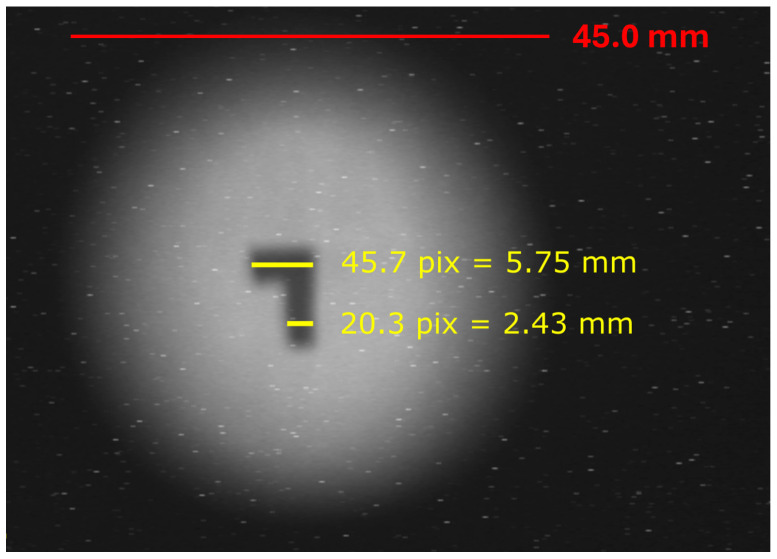
VESUVIO beam profile at thermal energies, obtained via a CCD camera and a neutron-absorbing converter material. The L-shaped darker region at the center of the beam corresponds to the cadmium foil used to calibrate the beam size, with the conversion parameter indicated by the yellow lines. A red bar corresponding to 4.5 cm is given as the size of the beam penumbra. Figure from the instrument internal calibration (2016).

Over the last two decades, the VESUVIO spectrometer at the ISIS Neutron and Muon Source [1] has provided a suitable testbed for neutron irradiation experiments. VESUVIO is an example of a thoroughly characterized beamline, where a number of different techniques have been tested against the same radiation environment. The characterization of the fast neutron beam component was presented in Ref. [2], as based on soft errors induced in field-programmable gate arrays based on static random access memories, combined with neutron activation measurements with energy threshold above 5 MeV. An estimated neutron flux above 10 MeV of (5.82 ± 1.05) × 10^4^ n/cm^2^/s was obtained for a proton current of 180 μA at a proton energy of 800 MeV. To evaluate the neutron fluence, the authors assumed a beam diameter of 4.5 cm, based on internal neutron-induced auto-radiographs. Rebai et al [3] provided a beam size evaluation for neutrons above 5.7 MeV using diamond detectors, obtaining (at 12.5 m from the moderator) values of Half Width at Half Maximum (HWHM) ranging between 1.8 and 2.3 cm. In 2016, a photograph of the neutron beam at the sample position was acquired as a function of a cut in neutron energy from 5 MeV to 20 MeV, with a neutron-gamma converter foil and a CCD camera, therefore biased towards the thermal neutron component, and showing an overall penumbra diameter of 4.5 cm, as shown in Figure 1. More recently, the thermal-to-epithermal neutron beam at the sample position (11.0 m from the moderator) was characterized using GEM detectors and neutron time-of-flight (TOF) [4], and a beam shape composed of an umbra and penumbra regions was defined, with diameters of 3 cm and 5 cm, respectively, approximately constant up to 100 eV, and later benchmarked by McStas neutron transport simulations in Ref. [5], which however provided a beam profile that was less flat and more Gaussian-like shaped.

The combination of this variety of techniques allows characterizations above nuclear-reaction thresholds, at several MeV energies and below hundreds of keV, with TOF and absorption processes. At intermediate energies, typical TOF experiments would provide only rough measurements, either because one relies on absorption processes for neutron detection, which become less efficient as one increases the neutron energy, or because the time resolution in the TOF measurement worsens. In the case of VESUVIO, all neutron energies above 50 keV are measured within the first 5 μs of TOF acquisition, which are generally discarded because of the high gamma-ray background within the experimental blockhouse, possibly causing detection saturation. Moreover, in the case of ISIS, the spallation process is based on two subsequent proton bunches hitting the target over a period of 0.5 μs, resulting in inaccuracies in converting TOF to neutron energies for such short TOF values. On the other hand, such intermediate energy is also responsible for a conspicuous portion of the overall neutron beam on VESUVIO. In fact, Bedogni et al. [6] provided a characterization of the neutron energy spectrum using Bonner spheres, and showed that about 11.5% of neutrons at the sample position had energies between 100 keV and 10 MeV, and 0.7% had energies above 10 MeV.

Solid-state nuclear track detectors (SSNTDs) [7] are materials that can be used to measure the dose released by charged particles through the damage induced in the polymer structure. Such damage, at the atomic scale, needs to be amplified and fixated through chemical etching. The etching technique and recipe can vary depending on the detector, as well as on the incident particle type and energy. In general, this technique relies on the etching being faster along the track than on the surface. In the case of neutron radiation, the projectile does not damage the detector directly, but it can induce a recoil of its constituent nuclei (H, C and O), as well as (n, α), (n, p) and (n, d) reactions in O and C. Among the plastic materials used, the most popular is CR-39; the commercial name for the polymer poly(allyl diglycol carbonate) (PADC), suitable dosimeters for neutrons [8] and protons with energies as low as 20 keV [9,10,11,12,13,14], although research and standardization in their use are ongoing processes [15]. As individual personal dosimeters, CR-39s have been extensively studied through experiments and computer simulations, as well as etching criteria [16,17,18,19,20,21]. Historically, CR-39 dosimeters have been used in applications with sources of fast neutrons, peaked at certain MeV energies, such as α-induced or fission sources [22,23,24,25,26,27,28]. Although they have been reported to be sensitive to neutron energies as low as 100 keV, characterizations with epithermal-to-intermediate neutron fluxes are missing, and could be useful for further benchmarks of CR-39 responses [29].

Here, we provide a beam-shape characterization and direct visualization of the VESUVIO neutron beam, in the intermediate energy region between 100 keV and 10 MeV, using CR-39 dosimeters. The choice of this type of dosimeter was suggested by its minimum detection energy, similar to the maximum neutron energy allowing TOF characterizations (e.g., as in Ref. [4]), yet significantly lower than the nuclear-reaction threshold energies used in diamond-like detectors (e.g., as in Ref. [3]). Moreover, this class of plastic dosimeters approximately reproduce human body interactions with epithermal-to-fast neutrons, owing to their hydrogen-rich chemical composition. The radiation weighting factor of neutrons gives a significant (about eight-fold) increase in the energy range between 100 keV and 10 MeV compared to thermal or faster neutrons [30], which is closely related to the recoil of hydrogen nuclei and their dose-related relative biological effect. In this framework, a precise characterization of neutron beams at these intermediate energies could be an important piece of information for user communities interested in radiation protection and medical physics.

## 2. Materials and Methods

### 2.1. Neutron Irradiation Experiments

CR-39 dosimeters, produced by Track Analysis Systems Ltd. (TASL) (Bristol, UK), were irradiated at the VESUVIO beamline [31]. The dosimeters, composed of the polymer PADC, which contains C, H, and N at natural isotopic abundances, had an area of 2.5 × 2.5 ${\mathrm{cm}}^{2}$ and thickness of 1 mm, and were therefore irradiated by placing them, four at a time, in a 2 × 2 matrix perpendicular to the neutron beam, so as to cover an overall area of approximately 5 cm by side. Each dosimeter featured an engraved serial number and area marker, to be used for spatial calibration of images. Multiple groups of dosimeters were irradiated at several irradiation times: 5, 10, 30, and 60 min. During the irradiation measurements, the stability of the neutron beam was monitored using the incident neutron monitor, providing an effective neutron count for each measurement. The nominal proton current in the ISIS synchrotron was 170 μAh. Additional measurements [32] were performed at the NILE neutron generator at ISIS [33], where deuterium-tritium reactions generate a neutron yield of 1.2 × 10^9^ neutrons/s. By placing the dosimeters at a distance from the generator of 65 cm, a fast neutron flux of 2.3 × 10^4^ neutrons/cm^2^/s was estimated, corresponding to a fluence of 2.7 × 10^6^ neutrons/cm^2^ after an irradiation of 2 min. Prior to the neutron experiments, all dosimeters were inserted in sealed polyethylene bags at the laboratory in Rome, to minimize radon exposure. Moreover, the set of irradiated dosimeters were accompanied by a reference sample that was not irradiated, providing a background reference related to exposure to environmental radiation related to flight and security control while traveling from Italy to the UK. We performed measurements with and without a PMMA radiator of thickness 1 mm, without observing significant differences, substantiating that the majority of detected events, up to 10 MeV neutron energy, were due to neutrons creating recoiling ions within the CR-39 material, as discussed in [34].

### 2.2. CR-39 Etching Procedure

**Figure 2:**
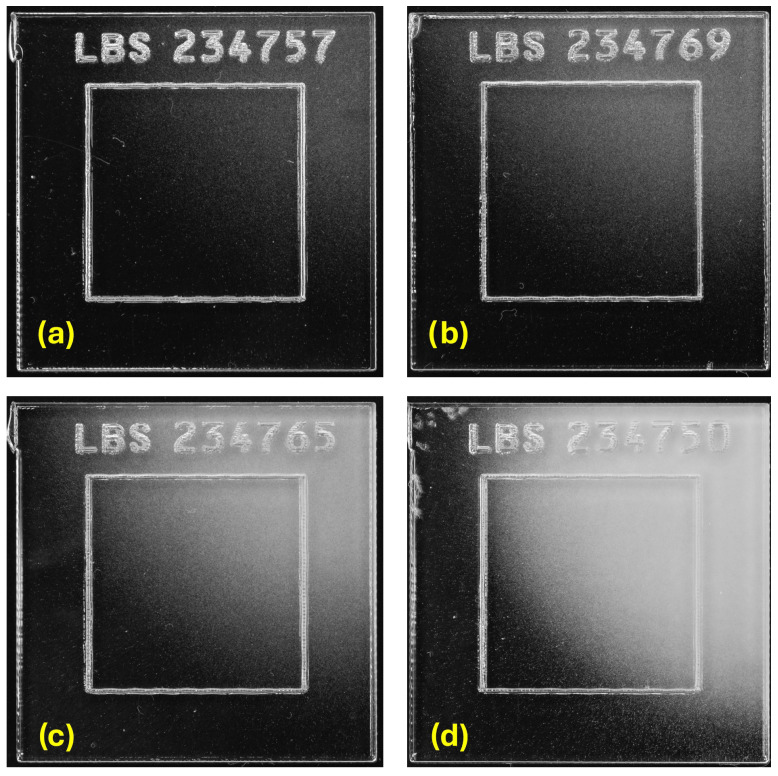
CR-39 dosimeters (after etching) irradiated at VESUVIO for 5 min (**a**), 10 min (**b**), 30 min (**c**), and 60 min (**d**).

After irradiation, all the dosimeters underwent the etching process, in order to develop visible tracks. The procedure consisted in a pre-etching phase for 30 min at a temperature of 70 °C in a solution of 60% NaOH (6.25 N) and 40% ethanol. In a second phase, the etching was performed over a period of 70 min at a temperature of 97 °C in a NaOH solution (6.25 N).The dosimeters also showed, macroscopically, partial saturation with the longer irradiation times in the spatial region where the neutron beam center was expected. This enabled performing a simple inspection of the neutron beam shape, particularly for the dosimeters irradiated for 30 and 60 min, as shown in Figure 2.

Depending on the etching time and protocol, tracks may appear with varying shapes and sizes. For charged heavy particles, it is possible to define a critical angle, which represents the smallest angle to which the particle that enters the detector can be visualized after etching. This angle depends on the bulk etching velocity $V_{B}$ and the track etching velocity $V_{T}$, and can be calculated as in [7]:

(1) $$\theta_{c}=\arcsin\left(\frac{V_{B}}{V_{T}}\right).$$

The diameter and length of the track are given by the velocities of bulk and track, namely $L=(v_{T}-v_{B})t$ and $D=2v_{B}t\sqrt{\left(v_{T}-v_{B}\right)/\left(v_{T}+v_{B}\right)}$ [26].

However, in the case of neutrons, the point of origin of the charged ions (H, C, and O) is expected anywhere within the bulk, especially at epithermal to fast neutron energies, which correspond to neutron mean free paths comparable to or longer than the dosimeter thickness. Therefore, one can expect polymer damage at any distance below the surface layer, and the bulk etching is not expected to bias the measurement. Secondly, depending on the etching time, an approximately linear original track can become a conical or spherical defect in the etched dosimeter.

### 2.3. Analysis of Optical Images

Images of the dosimeters were acquired with a Canon 5d Mark III, with a Canon 100 Macro Objective, with one image per dosimeter. Only dosimeters with 30 and 60 min irradiation times were analyzed, in order to have a strong neutron-beam signal in the grayscale compared to other signals, such as the one from the border incisions on the dosimeters. Each dosimeter was processed by applying a Gaussian blur to uniform the grayscale, especially where tracks were more sparse, using a standard deviation for the Gaussian blurring of 0.05 cm. Then, each image was normalized to the full grayscale (0–255), imposing the maximum and minimum values as the average value of two regions of interest chosen to be representative of the gray value of the maximum and minimum of the curve. Specifically, the maximum value was estimated as the average of a region located in the center of the beam, while the minimum value was set as the average of a dark region, on the side of the image. To test the enhanced polar symmetry of the beam shape, the processed images were then analyzed using the Hough Circle transform, to detect the center and radius of the arc of circumference visible in each picture. The Hough transform requires setting a threshold on the grayscale: the same measurement was performed at multiple thresholds, chosen to correspond to a percentage attenuation of the maximum intensity. Due to uncertainties in the grayscale, caused by the incisions in the dosimeters, the optimal threshold was found to be around a 70% attenuation of the maximum intensity.

All images were well fitted using circles as a model function, confirming the circular nature of the beam, as already assessed in previous characterizations. The parameter of the Hough transform known as the accumulation step, which defines the resolution of the transform, was defined to correspond to a spatial dimension of 0.01 cm.

### 2.4. Scanning Electron Microscopy Characterizations

Scanning Electron Microscopy (SEM) was performed using a TESCAN VEGA available at the ISIS@MACH ITALIA research infrastructure, equipped with a tungsten filament source and a four-lens Wide Field Optics column. SEM images were collected using Everhart–Thornley Secondary Electrons (SE) and a back-scattered electrons (BSE) detector, whereby an X-ray detector was employed for Energy Dispersive X-ray Spectroscopy (EDS) microanalysis (Oxford Instruments INCA 200). Samples were coated with gold to reduce electrostatic charging during SEM acquisition. Measurements were performed in vacuum (10^−6^ mbar).

### 2.5. Monte Carlo Simulations

To quantify the track formation length, before etching, we performed Monte Carlo transport simulations using the Geant4 toolkit, version 10.7.4., employing the physics list QGSP_BIC_HP. This is a reference physics list available inside the toolkit, which combines the models of the QGSP_BIC reference physics list, used for the simulation of precise hadronic interaction, with the models for “high precision” (HP) neutron interaction, which take into account neutrons with energies below 20 MeV [35,36]. Targets with the same physical dimensions of the actual CR-39 dosimeters were placed in a vacuum and irradiated with a monochromatic neutron beam perpendicular to the flat dosimeter face, covering its entire area and neglecting any beam divergence. Therefore, at the detector position, the beam area has the same dimensions as the dosimeter itself. The PADC material was implemented as a custom-defined Geant4 material, with stoichiometry C_12_H_18_O_7_ and density 1.15 g/cm^3^. A series of simulations were carried out for different energies of the primary monochromatic neutrons (10, 20, 50, 100, 200, 350, 435, 500, 750, and 900 keV; as well as 1, 1.2, 3, 5, 10, 14.1, 50, and 100 MeV). Scoring was implemented through a custom Geant4 sensitive detector and hit classes, which recorded the secondary particle type, as well as its energy deposition and step length for each step inside the detector, accumulating these values to obtain the total deposited energy and track length. These secondary particles are the natural isotopes of hydrogen, oxygen, and carbon that are present inside the CR-39 material, which travel inside the dosimeter after neutrons induce their recoil. At MeV energies, secondary particles could also include products of inelastic nuclear reactions on the sample isotopes.

The number of tracks was estimated using several thresholds. The smallest threshold was set equal to the step length in the simulation trajectories of charged particles. The step length was manually decreased to either 1 nm or 2 nm.

## 3. Results and Discussion

### 3.1. Beam Profile with CR-39

**Figure 3:**
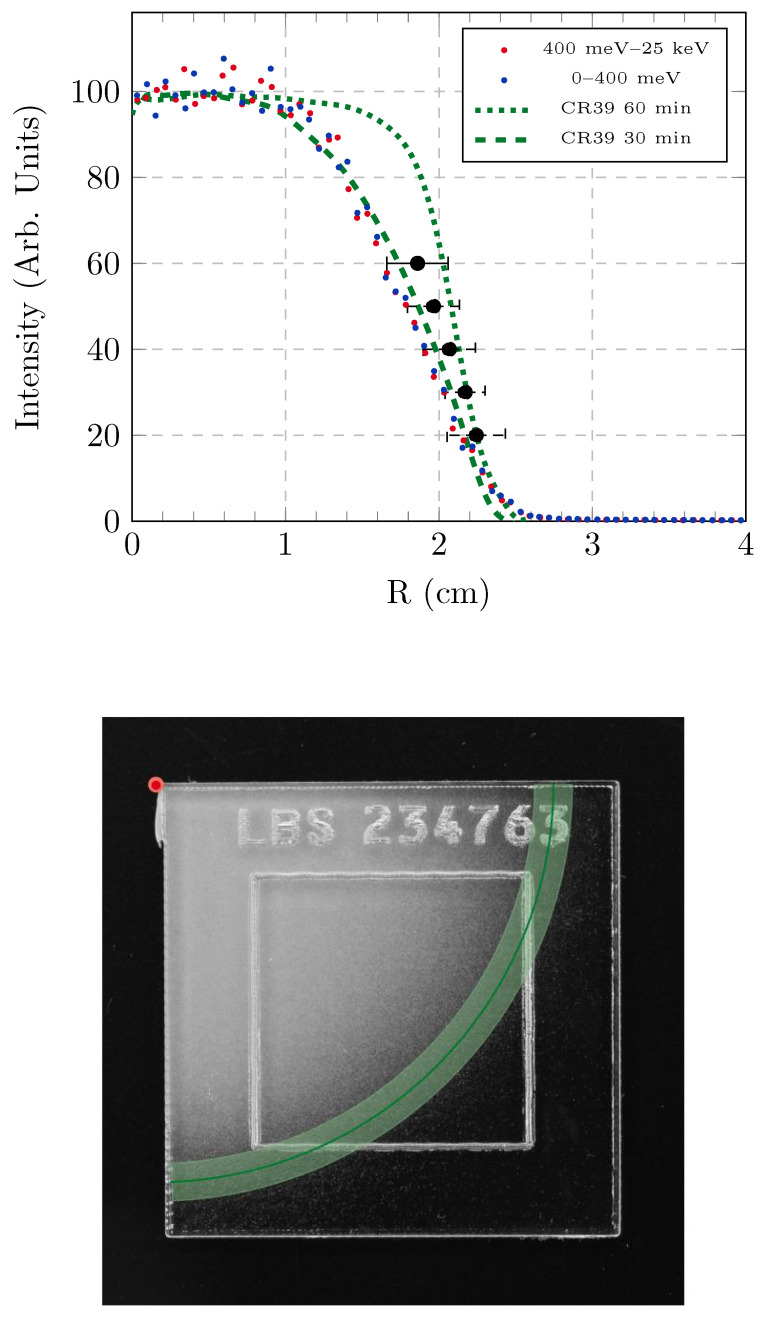
(**Top**) The intensity profile for the VESUVIO spectrometer obtained by analyzing CR-39 dosimeters was compared to experimental and literature intensity profiles reported in [4,5]. The dashed and dotted curves represent the intensity profile of two CR-39 dosimeters, irradiated for 30 and 60 min, respectively. All the curves are normalized to a maximum intensity of 100. It is possible to see the effect of saturation on the shape of the curve. The black dots and their error bars represent the mean and standard deviation obtained by analyzing the irradiated dosimeters with the Hough circle transform, at different attenuation percentages. (**Bottom**) An example of the analysis based on the Hough circle transform, showing an arc of a circle corresponding to a radius of 2.17 ± 0.13 cm obtained with a threshold of 70% attenuation, superimposed on an optical image of one of the dosimeters irradiated on VESUVIO.

Figure 3 shows the neutron beam profile of the VESUVIO spectrometer obtained by analyzing the CR-39 dosimeters irradiated for 30 min and 60 min. In both cases, the beam profile was found to be circular, as anticipated by visual inspection of Figure 2 and resulting from the analysis based on the Hough transform. The profile associated with the longer (60 min) irradiation time is broader and with a flatter-top, although it has similar dimensions for the beam penumbra, with the beam intensity larger than 1% of the maximum intensity up to 2.5 cm of radius. The black error bars in the Figure represent the value of the average radius, corresponding to a given threshold level in the Hough transform analysis, for maximum-intensity attenuation levels between 40% and 80%.

**Figure 4:**
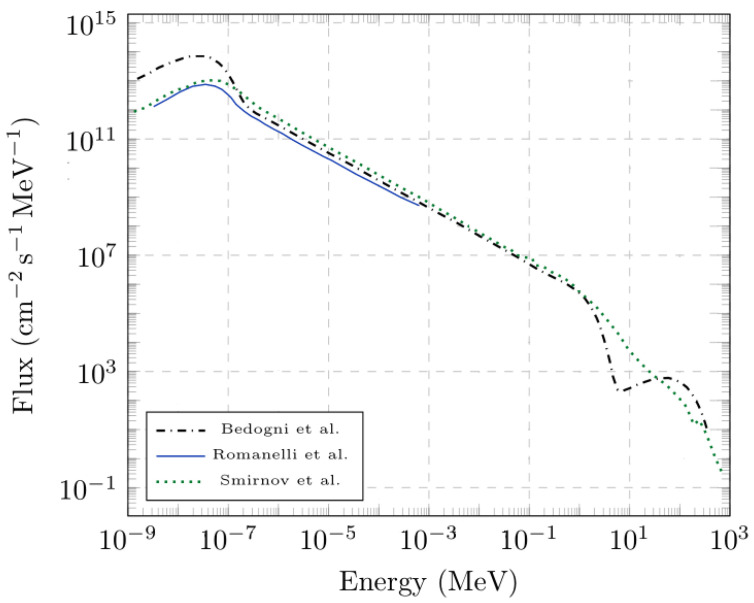
VESUVIO beam flux as measured with Bonner spheres (Bedogni et al. [6]), with TOF using a GEM detector (Romanelli et al. [4]), and with fission chambers (Smirnov et al. [37]).

The results from the present study were compared with the experimental results reported in [4], which are shown here after integration up to 400 meV and between 400 meV and 25 keV, near the technical limit of TOF measurements on VESUVIO. One can appreciate the agreement between the beam profile characterized by the CR-39 irradiated for 30 min and the profiles obtained from previous TOF measurements at thermal and epithermal energies. This result can be explained by the geometry of the incident VESUVIO flight path, facing the room-temperature water moderator. The resulting beam flux, reported in Figure 4, is composed of a Maxwell–Boltzmann distribution peaked at about 25 meV, with a long 1/E tail in the epithermal region and up to about 1 MeV, and a final drop to about 700–800 MeV, which is the nominal energy of protons producing neutrons in the target by spallation. Between the moderator and the blockhouse, a series of collimation slits were found to modify the shape of the thermal neutron beam from square, due to the square-shaped face of the moderator, to circular [4,5]. The transport of neutrons between the moderator and the sample position is, therefore, only governed by absorption and removal processes, as there are no neutron-optics elements, such as guides, in the primary flight path on VESUVIO. Considering the amount of absorbing material, as well as the approximately circular profile that was also found for fast neutron characterizations with diamonds [3], it is more than reasonable that the beam profile for intermediate-energy neutrons is the same as for epithermal ones (up to 25 keV).

The comparison of our results with literature data, in Figure 3, prompts the conclusion that the broader and flatter beam profile obtained by neutron irradiation of CR-39 dosimeters for 60 min may be associated with saturation effects. Saturation of tracks after etching, as seen by optical probes, contributes to a completely overlapping signal over a broader range of dimensions. In the outer region of the neutron beam, where the flux is lower, the agreement between the signal from 30-min and 60-min exposure times improves. In the case of the Hough-transform analysis, one should note that the average and standard deviations, resulting in the black markers and error bars in Figure 3, were obtained from a global analysis including both irradiation times, and are therefore expected to provide the most accurate results at higher attenuation values of the maximum intensity.

### 3.2. Track Analysis Using Scanning Electron Microscopy

**Figure 5:**
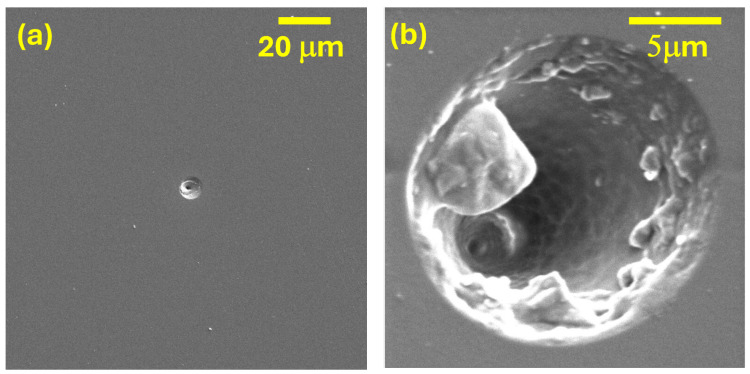
SEM images of an irradiated CR-39 dosimeter using the NILE D-T neutron generator (14 MeV) at 2000 magnifications (**a**), 15.000 magnifications (**b**) and 5 keV of incident electron energy.

**Figure 6:**
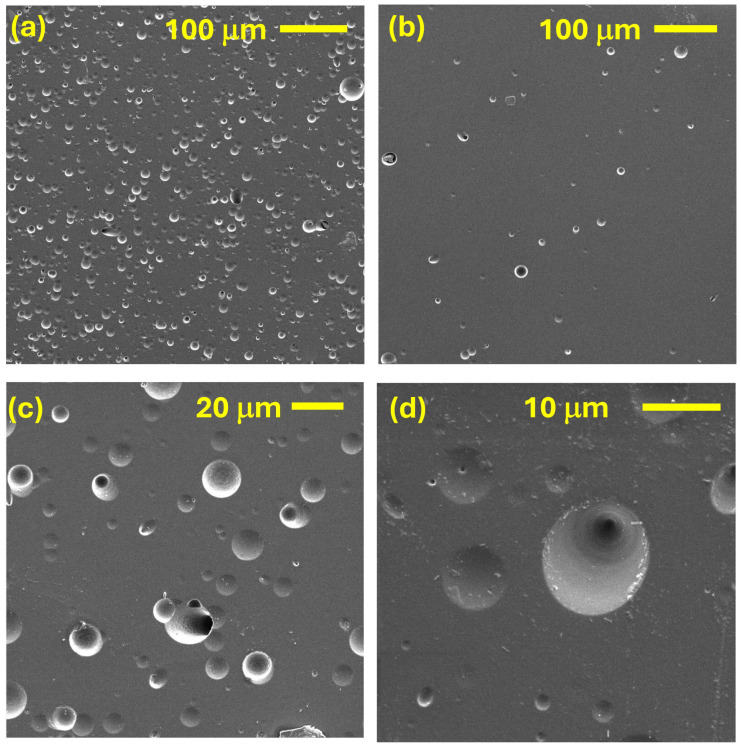
SEM images of an irradiated CR-39 dosimeter on VESUVIO in a region near the beam center (**a**) and in a region away from the beam center (**b**). Two images at higher magnification of intermediate regions are also shown in (**c**,**d**).

As mentioned, the irradiation of the CR-39 dosimeters on VESUVIO, and the subsequent etching procedure, lead to track saturation, especially at the center of the neutron beam. As this prevented us from analyzing the larger tracks through optical microscopy, SEM characterizations of the samples were performed, both in the case of dosimeters irradiated on NILE (e.g., those in Figure 5) and on VESUVIO (e.g., those in Figure 6).

First, one should note that the spatial distribution of the tracks is expected to be markedly different on the two instruments: Nile produces fast neutrons in all directions, resulting in a homogeneous distribution of tracks over the dosimeter surface. Considering the small surface of the dosimeter, compared to the distance from the neutron source, one can approximate the incident neutron trajectories as perpendicular to the dosimeter surface. On the other hand, on VESUVIO, the density of tracks is expected to be much higher near the beam center and relatively low towards the outer regions. This can be qualitatively appreciated by comparing panels (a) and (b) of Figure 6.

**Figure 7:**
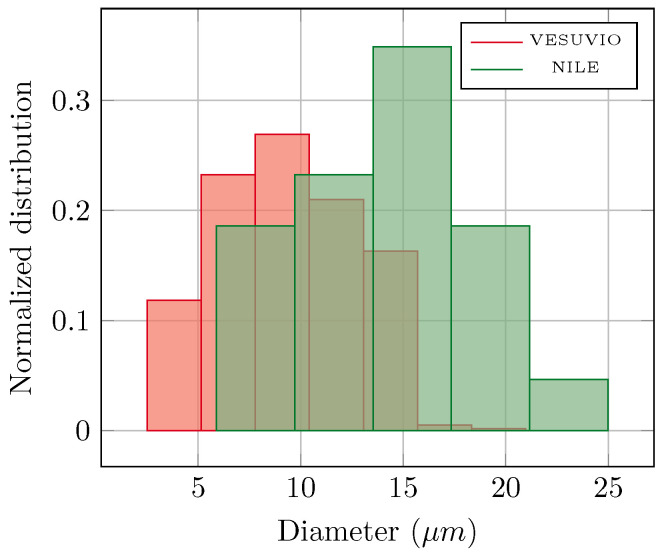
The distribution of the fitted values of the track diameters as obtained from the SEM images of neutron-irradiated dosimeters at the VESUVIO and NILE beamlines. Due to limited available data, the NILE track distribution histogram was constructed with only 5 bins, in contrast to the 7 bins used for VESUVIO.

In principle, one could also expect a significant difference in the track dimensions, as on VESUVIO one has a broad-energy neutron beam (Figure 4), while on NILE, under D-T operations, one has an almost mono-energetic beam with 14 MeV neutrons. However, this comparison is made more difficult due to the several resolution and convolution components that link the original neutron energy and the final track length. First, nuclear recoil induced by epithermal-fast neutrons can happen at several angles, thus resulting in a distribution of nuclear-recoil energies, each resulting in a different track length. Second, the etching process will concurrently remove a bulk layer of material, with a given velocity, as well as travel along the track and amplify its dimension, with a different velocity. The resulting track shape and dimension will therefore be related to the original point of the track with respect to the original (pre-etching) surface of the dosimeter. Notwithstanding these considerations, Figure 7 shows the normalized distributions of track lengths as obtained by the fitting of the SEM images from several CR-39 dosimeters irradiated on VESUVIO and NILE. One can observe that the VESUVIO distribution is shifted to shorter track diameters compared to NILE, as one would expect.

Moreover, it is clear from the comparison of Figure 5 and Figure 6 that a markedly higher number of tracks were produced on the dosimeters irradiated on VESUVIO. We performed a count of the track occurrence over a set of SEM images corresponding to VESUVIO-irradiated (5 min, near the beam center) and NILE-irradiated dosimeters (2 min), and we obtained track densities of 1950 ± 66 tracks/mm^2^ for VESUVIO and 9.4 ± 2.7 tracks/mm^2^ for NILE, providing a ratio of 207 ± 60. The independent field of views analyzed to obtain these results were 6 and 18, for VESUVIO and NILE, respectively. This result can be compared to estimates based on the known beam fluxes on VESUVIO and NILE. A rough comparison can be obtained by taking the ratio of the total fluence of fast neutrons in the two cases. On VESUVIO, integrating the beam flux from Smirnov [37], one finds that the fast neutrons with energies between 10 MeV and 100 MeV integrated over 5 min of irradiation are 2.2 × 10^7^ neutrons/cm^2^ (which would increase to 2.4 × 10^7^ neutrons/cm^2^ integrating up to 800 MeV), while the number of 14-MeV neutrons on NILE over 2 min is 2.7 × 10^6^ neutrons/cm^2^, providing a ratio of about 8, much smaller than the SEM measurement (about 200). To explain such a difference, suggesting that the largest part of the tracks is related to neutrons with energies below 10 MeV, we performed Monte Carlo simulations, as discussed below.

### 3.3. Interpretation Through Monte Carlo Simulations

**Figure 8:**
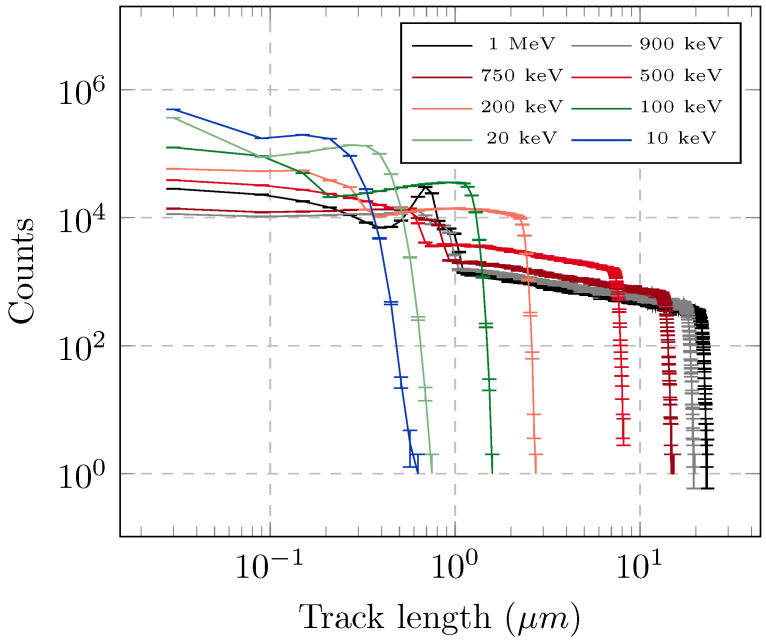
The number of tracks produced by monochromatic beams of $10^{7}$ neutrons at several incident energies as a function of the track length.

**Figure 9:**
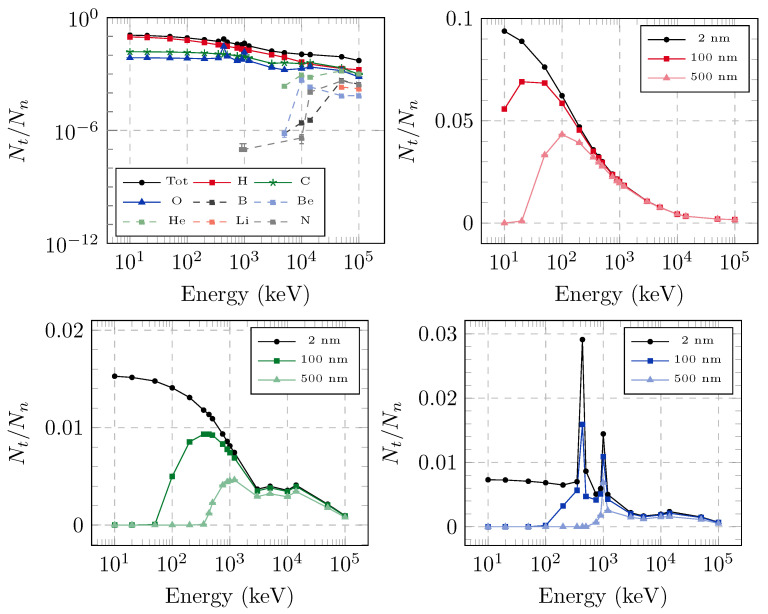
(**Top left**) The total number of tracks (longer than 2 nm) per incident neutron of a given incident energy (black dots), as well as the individual contribution from all secondary nuclei detected. The other panels report the number of tracks longer than 1 nm, 100 nm and 500 nm, generated by hydrogen (**top right**), carbon (**bottom left**), and oxygen nuclei (**bottom right**). It is interesting to note that the peaks in the latter plot appeared at energies that correspond to elastic resonances in the neutron cross-section of oxygen.

Geant 4 Monte Carlo simulations were performed for multiple energies of the incident neutron beam, ranging from 10 keV up to 100 MeV. Simulations were interrogated for the number of tracks per primary neutron, as a function of both the track length and the secondary particle associated with the track. The number of tracks with different lengths is reported in Figure 8, for different energies of incident neutrons, while Figure 9 shows the number of tracks per primary neutron, due to specific secondary nuclei, above several thresholds for the minimum track length.

The step length used in the Monte Carlo simulations is of the same order of magnitude as the size of a monomer, and it is not useful to further reduce the minimum step, as the presence of scattering centers is discrete and highly inhomogeneous in reality, as opposed to the standard assumption in Monte Carlo simulations of a homogeneous medium. However, such small tracks are not expected to result in etched marks, as they do not allow for chemicals to diffuse along the broken polymer. For this reason, further physical thresholds of 100 nm and 500 nm were also considered as minimum track lengths that could result in detectable defects on the etched surface. The number of tracks and their lengths were influenced by multiple phenomena, each modeled by a cross-section in the Monte Carlo code. As an example, it is possible to see the higher number of produced oxygen tracks at the approximate energies of 400 keV and 1 MeV: this was due to (n, n) resonances in the neutron-oxygen cross-section, peaking at about 1 MeV. The probability of interaction of the secondary particles with the polymer atoms also played a role, causing a slight increase in the number of tracks even where there were no resonances in the neutron cross-section. Moreover, secondary particles can also induce recoil, causing a cascade production of tracks, for each incoming neutron. For these reasons, Monte Carlo transport simulations prove to be a useful tool to study such a complex system.

Our results for the response function are in agreement with those reported for the CR-39 latent efficiency in Refs. [38,39], for H, C, and O, as well as for their sum. Moreover, the total latent efficiency therein, more closely related with the total number of tracks rather than to the dose deposited in the dosimeter (compared e.g., with Ref. [21]) shows an almost continuous decrease moving from 500 keV to 14 MeV, only partly affected by the presence of resonances or threshold nuclear reactions. However, in both Figure 8 and Figure 9, the number of tracks is calculated over the entire volume of the CR-39 dosimeter. While they are homogeneously distributed within such a volume, considering the penetration length of the incident neutrons, they are not easily scaled to surface information, i.e., the number of defects detectable on the final etched surface. In particular, one can expect that shorter tracks further away from the final etched surface will provide defects that are hardly visible, as opposed to longer tracks nearer to the final surface.

**Figure 10:**
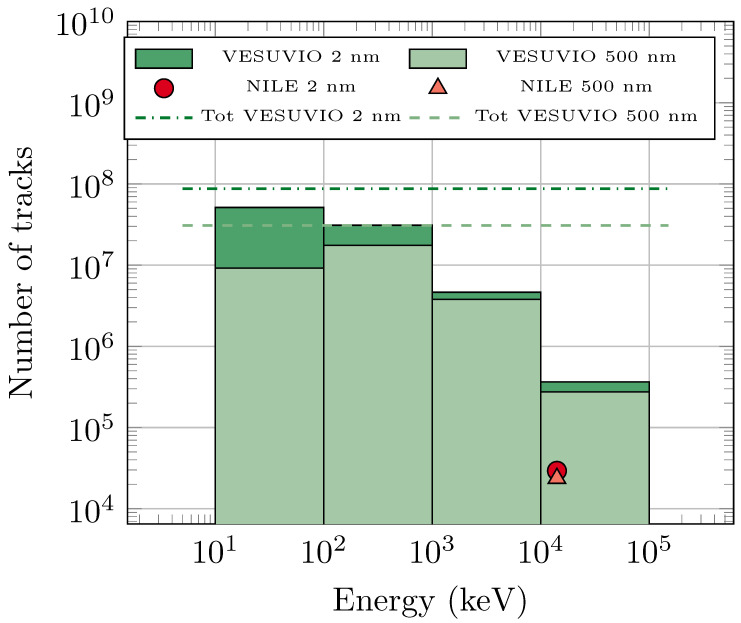
Number of tracks expected from simulations after a 5 min VESUVIO irradiation from several neutron energy intervals (histogram), as well as the total (dashed line), with track length thresholds at 2 nm and 500 nm. The number of tracks after 2 min irradiation on NILE is also reported as markers.

Despite the difficulty in linking the number of tracks in the dosimeter volume with the number of defects on the etched surface, one can still use the Monte Carlo result to qualitatively assess the main source of tracks on VESUVIO and explain the experimental SEM results. In this context, Figure 10 shows the overall number of tracks expected after a 5 min irradiation on VESUVIO, combining the response functions from Figure 8 and Figure 9, together with the actual neutron flux adopted from Ref. [37]. For each energy region, the histogram corresponds to the integrated number of tracks associated with the histogram bin. The results were calculated for length thresholds of 2 nm and 500 nm, and Figure shows, as horizontal lines, the total number of tracks obtained by summing the considered histogram counts. In the same Figure 10, the number of tracks calculated in the case of NILE and an irradiation time of 2 min is also reported.

First, one can see that the calculated ratio of tracks from neutrons with energies above 10 MeV on VESUVIO (5 min) is about an order of magnitude larger than on NILE (2 min), which compares very well with the estimate from the neutron fluxes provided earlier (factor of 8). On the other hand, when comparing the overall number of tracks on VESUVIO-irradiated dosimeters (dashed lines) with the number of tracks on the NILE-irradiated dosimeter, one obtains a ratio of about 1300, which is sensibly larger than that obtained from the estimate from the analysis of SEM images (factor of about 200). As mentioned earlier, the ratio of the number of tracks within a volume element is not expected to match the ratio of the number of defects on the etched surface, as not all tracks will be converted into visible defects. However, it is clear from the Monte Carlo simulations, with a physical length threshold, that more than half of the tracks were due to neutrons with energies between 100 keV and 10 MeV. Of the remaining contributions to the overall number of tracks in the CR-39 volume, the contribution from neutrons with energies above 10 MeV was substantially smaller, while the contribution from neutrons with energies below 100 keV is expected to be largely suppressed when converting to detectable defects on the etched surface.

## 4. Conclusions

We have provided an experimental determination of the VESUVIO beam profile using optical imaging analyses of etched CR-39 dosimeters. The beam shape obtained with this technique was in optimal agreement with previous determinations based on absorption processes and time-of-flight measurements. Additional analyses using scanning electron microscopy and Monte Carlo transport simulations suggested that the majority of the measured tracks could be related to neutrons with energies in the range between 100 keV and 10 MeV. Therefore, one can conclude that the VESUVIO beam profile, consisting of an umbra (intensity greater than 90%) with a radius of 1.1 cm and a surrounding penumbra (intensity greater than 1%) extending up to 2.5 cm, is the same for all energies from thermal neutrons up to 10 MeV. 

## Data Availability

The raw data supporting the conclusions of this article will be made available by the authors on request.
